# Arthritis with chronic pancreatitis—do not forget pancreatitis, panniculitis and polyarthritis (PPP) syndrome

**DOI:** 10.1093/omcr/omae203

**Published:** 2025-03-20

**Authors:** Ramsha Ansari, Deepanjali Golshetty, Vishrutha Poojari, Shivangi Tetarbe, Ira Shah

**Affiliations:** Department of Pediatric Gastroenterology and Hepatology, B J Wadia Hospital for Children, 3 Acharya Donde Marg, Mumbai, Maharashtra 400014, India; Department of Pediatric Gastroenterology and Hepatology, B J Wadia Hospital for Children, 3 Acharya Donde Marg, Mumbai, Maharashtra 400014, India; Department of Pediatric Gastroenterology and Hepatology, B J Wadia Hospital for Children, 3 Acharya Donde Marg, Mumbai, Maharashtra 400014, India; Department of Pediatric Gastroenterology and Hepatology, B J Wadia Hospital for Children, 3 Acharya Donde Marg, Mumbai, Maharashtra 400014, India; Department of Pediatric Gastroenterology and Hepatology, B J Wadia Hospital for Children, 3 Acharya Donde Marg, Mumbai, Maharashtra 400014, India

**Keywords:** pancreatic panniculitis and polyarthritis syndrome, PPP syndrome, pancreatic arthritis, chronic pancreatitis, pediatrics

## Abstract

Pancreatitis panniculitis and polyarthritis syndrome (PPP) syndrome is a rare complication of existing pancreatic disorders which include a triad of pancreatitis, oligo or monoarthritis and lobular panniculitis. We describe a case of a 3-year-old female child who presented with chronic pancreatitis and later developed knee pain during hospitalization. Other causes of arthritis were ruled out and pancreatitis-related arthritis, PPP syndrome was suspected. PPP syndrome treatment primarily involves managing the underlying pancreatic cause. In our case, managing the chronic pancreatitis with NSAIDs significantly improved the joint involvement.

## Introduction

Pancreatic diseases may complicate into pancreatitis, panniculitis and polyarthritis (PPP) syndrome [1]. PPP syndrome is a rare clinical syndrome consisting of an underlying pancreatic disease, panniculitis, poly or oligo-arthritis with intraosseous fat necrosis [1]. The main etiological factor leading to PPP syndrome is regarded to be an underlying pancreatic pathology, mainly acute pancreatitis (AP), chronic pancreatitis (CP) and pancreatic malignancies [2]. Other reported causes include ischemic pancreatic disease, abdominal trauma, pancreatic pseudocyst and pancreas divisum [3]. Around 2%–3% of patients with underlying pancreatic diseases are speculated to complicate into PPP syndrome [4]. The median age of presentation of PPP syndrome is around 49 years, indicating its rare occurrence in children [2]. We present a child with CP who developed knee pain after 4 months of abdominal pain indicating a possibility of PPP syndrome.

## Case report

**Figure 1:**
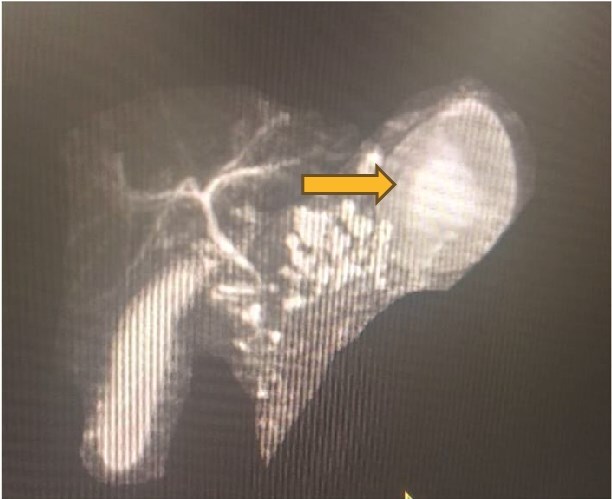
MRCP (magnetic resonance cholangiopancreatography) showing a moderately atrophied pancreas with T1 hypointense and T2 hyperintense collection seen in gastro splenic region abutting and indenting over greater curvature of the stomach. It measures around 3.2 × 6.4 × 3.9 cm, reaching anteriorly to the left lateral aspect of the liver and posteriorly to the splenic hilum. The main pancreatic duct (MPD) is irregular but non-dilated in the head, and MPD in the body and tail shows non-separate visualisation from the collection.

A 3 year 3-month-old female child, born of a non-consanguineous marriage, presented to us in December 2023, with severe generalised abdominal pain for 3 months, which increased with food intake and affected her daily activities. She also had non-projectile vomiting soon after meal intake. On presentation, her weight was 9.2 kgs (less than 3^rd^ centile according to WHO [World Health Organisation] growth charts) and height was 87 cm (less than 3^rd^ centile according to WHO growth charts). On abdominal examination, she had epigastric fullness. Other systemic examinations were normal. Serum amylase was 125 U/l and serum lipase was 118 U/l. Antinuclear antibodies were weakly positive (1:100), immunoglobulin G (IgG) was 1070 mg/dl (normal: 250–1600) and IgG4 levels were 1.16 g/l (normal < 1.4) (Table 1). The child was kept nil by mouth and given maintenance intravenous fluids for 24 h along with paracetamol and opioids. The child had persistent abdominal pain and could not tolerate oral feeds, for which an endoscopy-guided nasojejunal tube was inserted and kept for 4 weeks. Oral morphine was also given for persistent severe abdominal pain, which was titrated as per symptoms over 5 weeks. MRCP was performed (Figure 1). She was also given low molecular weight heparin for 20 days for retro-pancreatic portal vein thrombosis. Oral feeds were started after fifth week of hospitalisation. Nutritional rehabilitation with high-calorie diet was started. Genetic study was not performed due to financial constraints.

**Table 1:** Laboratory findings of our patient.

**Investigation**	**7 days prior to admission**	**Day 1 of hospitalization**	**Day 6 of hospitalization**	**Day 16 of hospitalization**	**Day 19 of hospitalization**	**Day 30 of hospitalization**	**Day 37 of hospitalization**	**Normal range**
Haemoglobin (gm/dl)		9.5	-	7.7	10.7	-	9.3	11.5–15.5
White cell count (cells/mm^3^)		10 720	-	7930	7330	-	16 640	5000–13 000
Platelets (10^6^ cells/mm^3^)		6.61	-	8.36	6.66	-	7.48	1.5–4.5
Serum amylase (U/l)		125	-	-	-	-	-	40–140
Serum lipase (U/l)		118.3	-	-	-	-	-	10–140
C3 (mg/dl)		-	-	-	-	191	-	75–175
ASLO		-	-	-	-	Non-reactive	-	
ESR (mm/h)		-	-	-	-	64	30	< 10
CRP (mg/l)		-	2.8	8.36	14.6	-	5.6	< 3
Triglyceride (mg/dl)		-	164	-	-	-	-	
HDL cholesterol(mg/dl)		-	286	-	-	-	-	
VLDL cholesterol (mg/dl)		-	32.6	-	-	-	-	
Serum sodium (Meq/l)		135	139	135	-	-	139	
Serum potassium (meq/l)		4.0	3.6	4.9	-	-	5.0	
USG abdomen	3.0 × 1.8 × 1.7 cm (5 cc) collection in lesser sac. Normal pancreas.	3.3 × 2.6 × 3.5 cm (16 cc) collection in lesser sac. Pancreas could not be seen						
CT abdomen			Bulky heterogenous pancreas with multiple non enhancing hypodense lesions. 7.4 × 3.4 × 4.9 cm retro gastric collection and 1.1 × 1.4 cm collection posterior to MPV				Decreased number of collections with distorted head of pancreas. Resolved or ruptured retro gastric collection with a 1.3 × 1.6 cm pseudocyst posterior to body of stomach	
MRCP				Moderately atrophied pancreas with intercommunicating capsulated locules of collection. 3.2 × 6.4 × 3.9 cm retro gastric collection				

Note: CRP—C reactive protein, C3—Complement 3, ASO—Anti Streptolysin O, ESR—Erythrocyte sedimentation rate, HDL—High density lipoprotein, VLDL—Very low density lipoprotein, USG—Ultrasonography, MPV—Main portal vein, CT—Computed Tomography, MRCP—Magnetic resonance cholangiopancreatography.

On 4^th^ week of admission, the child developed a swelling in the right knee joint with redness of skin over the joint, mild restriction of movement and inability to bear weight in the right lower limb. She also had a right index finger swelling with skin redness at the proximal interphalangeal joint. On evaluation, anti-streptolysin O (ASO) titres were non-reactive. Complement 3 (C3) levels were 191 mg/dl, C-reactive protein was 14.6 mg/dl, and Erythrocyte sedimentation rate was 64 mm/h. X-ray and ultrasonography (USG) of both the knee and hip joints were normal. She was treated with non-steroidal anti-inflammatory drugs (NSAIDs) for 5 days, after which swelling and pain subsided. She was discharged on day 54. On follow-up, the child had occasional abdominal pain with poor weight gain. Abdominal USG after 4 months revealed a hypoechoic collection of size 4.7 × 1.9 × 3.5 cm, 16 cc volume, with echoes arising from the head and uncinate process of pancreas with rest of pancreas showing a bulky and heterogenous echotexture. The child was advised admission for nutritional rehabilitation with pancreatic enzyme replacement and fat-soluble vitamins, but the child was later lost to follow-up.

## Discussion

The pathophysiology of PPP syndrome remains unclear, however, it is believed that pancreatic enzymes released by the diseased pancreas, are responsible for the fat necrosis in the subcutaneous fat, bone and joints [2, 3]. It is speculated that trypsin and phosphorylase may damage the adipocytes adjacent to capillary endothelium to allow lipase to cause lipolysis and inflammation leading to necrosis of fat cells [3]. Some authors speculate that lipolytic pancreatic enzymes hydrolyse the triglycerides periarticular adipocytes forming free fatty acids, which deposit in the joints causing arthritis [5]. The diagnosis of PPP syndrome is usually made clinically. It is highly suggestive when there is presence of high lipase levels with skin or joint changes consistent with pancreatic panniculitis or pancreatic arthritis [6].

Pancreatic panniculitis presents as painless or painful oedematous, red-brown nodules typically over the distal parts of lower extremities and spread all over with disease progression [1]. These nodules can ulcerate or rupture to drain a sterile, oily brown discharge from the fat necrosis. On histology, these lesions show lobular panniculitis without vasculitis. A pathognomic finding seen are ‘ghost cells’, which are necrotic adipocytes [3]. Arthritis in PPP syndrome is usually symmetric polyarthritis commonly in distal extremities like ankles, hands and knees, but it can also present as monoarthritis or oligoarthritis [1]. Synovial fluid analysis shows sterile, thick fluid with high neutrophil count, high lipase and signs of chronic inflammation [1, 2]. Approximately 50% patients with PPP syndrome present with gastrointestinal symptoms, and their absence or delayed onset can potentially lead to misdiagnosis [3]. Other signs include chondronecrosis, necrosis of intramedullary fat and gastrointestinal mucosa [1]. The triad in PPP syndrome can occur simultaneously and can precede one another, accounting for difficulty in diagnosis. The differential diagnosis for panniculitis includes erythema nodosum, subcutaneous abscess or infectious panniculitis. Joint involvement in PPP syndrome can be misdiagnosed as septic arthritis, gouty arthritis, rheumatoid arthritis or reactive arthritis [4]. In our patient, ASO titres and C3 levels were normal, ruling out other causes of arthritis.

Effective treatment of patients with PPP syndrome requires management of underlying pancreatic illness [6]. Panniculitis and arthritis related to PPP syndrome can be treated symptomatically with NSAIDs, corticosteroids or colchicine, but these agents have a poor response in 79% of patients [7]. Despite this, our patient responded well to NSAIDs. The management includes treating the cause of pancreatitis which can range from chemotherapy and surgical excision for carcinomas, stent insertion for pancreatic duct stricture or drainage of pseudocysts [3]. The outcome of patients with PPP syndrome is related to the resolution of the underlying pancreatic cause [2]. In the review of Betrains et al, resolution was reported in 55% of cases and mortality of 27% suggestive of poor prognosis [2].

## Disclosure for Support

No form of support has been received for this work.

## Consent

Informed patient consent was obtained from the parent of the child.

## Guarantor

Dr. Ira Shah is the guarantor for this manuscript.
